# Effects of Two TGfU Lessons Period on Game Performance, Knowledge and Psychosocial Variables in Elementary Physical Education

**DOI:** 10.3390/ijerph17103378

**Published:** 2020-05-12

**Authors:** José L. Arias-Estero, Pablo Jaquero, Ana N. Martínez-López, María T. Morales-Belando

**Affiliations:** Facultad de Deporte, UCAM Universidad Católica San Antonio de Murcia, 30107 Murcia, Spain; jaquero93@gmail.com (P.J.); ananuria.martinezlopez@gmail.com (A.N.M.-L.); mtmoralesbelando@gmail.com (M.T.M.-B.)

**Keywords:** sport pedagogy, TGfU, tactics, PETE, teaching–learning contexts, pedagogical models, physical activity

## Abstract

The purpose of the present work was to explore whether fourth-grade physical education students improved their game performance, knowledge and psychosocial variables with Teaching Games for Understanding (TGfU) to a greater extent after an eight-lesson period in comparison to a 14-lesson period. The study followed a mixed-methods approach in which the design consisted of a first assessment, a second assessment after Lesson 9 (eight-lesson period) and a third assessment after Lesson 16 (14-lesson period). A TGfU floorball intervention was carried out between assessments. The participants (*n* = 40) were in their fourth year of elementary education. According to students’ background and setting, we decided to implement three broad and interrelated strategies to enact the intervention built on the TGfU pedagogical features. Data were collected through Game Performance Assessment Instrument, knowledge questionnaire, enjoyment, perceived competence and intention to be physically active scales and semi-structured interviews. Quantitatively, Friedman’s χ^2^ was used to explore differences in the variables and Wilcoxon’s *Z* post-hoc comparisons were performed to determine: (a) first–second and first–third assessment differences; and (b) second–third assessment differences. Qualitatively, data were open and axial coded line-by-line and incident-to-incident in sub-themes. The quantitative results show no significant differences between the two periods (*p* > 0.05). However, there were improvements after both periods compared with the first assessment (*p* < 0.05). The qualitative information supported that the pedagogical strategies implemented could be key to explain the similarities between the two practice volumes. In conclusion, the amount of practice should not be considered as the only variable in the design of interventions with TGfU.

## 1. Introduction

Traditionally, physical education classes are characterised by short instructional units, mainly because of the multi-activity model, which remains the dominant approach in elementary education [1]. The number of lessons which should integrate an intervention unit may vary, depending on the complexity of the content, students and other psychosocial variables [2]. According to Teaching Games for Understanding (TGfU), greater time frames may be required in comparison to traditional ways of teaching–learning to develop complex decision-making and technical execution skills in game-play environments [3]. However, several TGfU studies reported improvements in game performance, knowledge and psychosocial variables after a short intervention of eight lessons or fewer (e.g., [4,5,6]). It seems fully justified to analyse whether a short instructional unit can be useful for teaching–learning using TGfU, furthermore considering that the Bunker and Thorpe proposal implicitly accepts the nature of the school timetabling and curriculum organisational arrangements [1].

TGfU was designed as a resistance to participants’ perceived dissatisfaction with the technique-based lessons [7]. This kind of lessons led to an environment characterised by less skilful students, low success experienced by a large percentage of learners, teacher-dependent performers and boring, uncontextualised drills. In contrast, the TGfU instructional unit essentially should consider four aspects [8]. First, students must experiment the modified game form with game structures adapted to them and accommodated to a wider range of ability levels. Second, students must reflect in order to understand what they did, what they should have done and why. Third, they must learn the technical execution contextualised in decision-making, considering the commonalities of similar games, playing as a members of teams. Finally, they must be physically and mentally involved in each task, playing an active role in relation to their peers. As a consequence, students self-construct their own knowledge thanks to the environment designed by teachers.

TGfU has been highlighted as an example of constructivist approaches to learning [8]. According to the constructivist theory, students could learn to build new conscious knowledge based on their initial knowledge while they try to actively understand their experiences related to the environment in which they occurred [9]. This view requires not just knowing how to perform some action but also analysing problems, planning solutions, evaluating the effectiveness of their actions and making judgments about the consequences of their actions in a social environment [10]. In comparison to the technique-based way of teaching games, the development of these higher-order cognitive skills within the game demands higher intervention volumes [8]. Consequently, the last game centred approach reviews recommended enacting longer instructional units [3,11]. Particularly, Miller [3]. (pp. 50–51) claimed “*an intervention volume of greater than eight hours appears to be a common cut point for greater positive support for the use of a game centered approach in the development of decision making and skill execution variables*”.

After making a review of TGfU articles written in English, from double-blind and peer-review journals and published until July 2019, there were 16 studies that enacted a TGfU instructional unit in school setting. Intervention exposure was between 4 and 32 lessons with a duration of approximately 1 h. Considering only the investigations with a short unit, the interventions had a positive association with 71.43% of tested game performance-related variables, 75% of knowledge variables and 90.91% of psychological variables [4,5,6,12,13,14,15]. Considering only the investigations with a long unit, the interventions had a positive association with 75% of tested game performance-related variables, 100% of knowledge variables, 100% of psychological variables and 50% of physical activity variables [10,16,17,18,19,20,21,22,23]. From the five studies that showed effects sizes, this effect size was large in three studies with a short unit [5,6,13] and between medium and small in two studies with a long unit [21,23]. Therefore, although long interventions seem to be slightly more effective, the differences between long-short units were small. These scarce differences in effectiveness between high-low volume interventions could be caused by the variety of pedagogical practices when using TGfU more than because of the unit length [1]. For example, while some TGfU studies with a long unit do not show teaching–learning implementation features (e.g., content and goal per lesson, questions, modified games, challenging tasks, lesson structure, the alignment between such elements based on the principles of play and verification procedures [16,19,20]), other authors implemented a short TGfU unit with such teaching–learning implementation features, showing positive results [6,17,23].

Given the relevance of the environment in the process of teaching–learning, it is necessary not only to focus on intervention volume as a key variable, but on the relationship among a multitude of interacting variables in such social situation [24]. In other words, there are many process variables that impact the teaching–learning practice [2]. Recent studies have highlighted the influence of practising at an appropriate level of difficulty together with other psychosocial variables, as well as the instructional alignment, on the efficacy of teaching–learning [4,10].

In summary, the scarce differences in effectiveness between high-low volume interventions and the multifaceted interaction among teaching–learning variables would support the use of short TGfU units, since the predominance of such instructional interventions in most physical education programmes. The purpose of this work was to explore whether fourth-grade physical education students improved their game performance, knowledge and psychosocial variables with TGfU to a greater extent after an eight-lesson period in comparison to a 14-lesson period, while playing floorball. The hypothesis was that there would be no differences between the 8- and 14-lesson periods in game performance, knowledge and psychosocial variables. The possible no differences between unit lengths were explored through the participants’ perceptions in order to go further trying to understand the quantitative results.

## 2. Materials and Methods

### 2.1. Design

The study followed a mixed-methods approach in which the design consisted of a first assessment, a second assessment after Lesson 9 (eight-lesson period) and a third assessment after Lesson 16 (14-lesson period, Table 1). A TGfU floorball intervention was carried out between first, second and third assessments. From first assessment to Lesson 9 (eight-lesson period), attack and defence of shot, pass, dribble and losing the mark were taught (Table 1), whereas, during Lessons 11–16 (14-lesson period), the teaching of attack and defence of shot, pass and dribble was repeated (Table 1). At each assessment (Lessons 1, 10 and 17), we collected quantitative data from students’ game performance (decision-making, skill execution, cover and support), knowledge and psychosocial variables (enjoyment, perceived competence and intention to be physically active). Furthermore, we evaluated qualitative data with the students and the teacher to record their perceptions of game performance, knowledge and psychosocial variables.

### 2.2. Participants and Settings

The children (*n* = 40; M_age_ = 9.44; SD = 0.45 years) were in their fourth year of elementary education. They were distributed into two classes, consisting of 13 boys and 27 girls in total. The socioeconomic status of the students attending the school was low-to-moderate. All participants took part in all the floorball lessons. However, none of the students had either played team games or had any previous knowledge or practical experience with the TGfU approach. The previous sport experiences of three students were limited to recreational swimming programmes. All the students, the parents and the teacher were informed of the protocol and blinded to the study aim. The parents and the teacher signed an informed consent form before the investigation and the students agreed to participate. The main author’s University Research Ethics Committee approved the study, which was performed in accordance with the Helsinki Declaration.

The school was located in a medium-sized city in southern Europe and was coeducational, state, urban and non-religious. In this school, which had a small physical education department, there were approximately 250 students. The school had an outdoor multisport court of 44 m × 24 m and four goals. Students received two 60-min physical education lessons per week. The school was selected because its physical education curriculum and teaching methods followed the technique-based lessons of: (a) warm-up; (b) skill practice; and (c) game.

In the country of the study, TGfU is not part of the physical education and teacher education curriculum. The regular physical education teacher had no experience of TGfU. Consequently, another teacher, who was familiar with and had practised TGfU, implemented the lessons. This teacher was 30 years old. He had five years of physical education teaching experience in different schools, in two of which he had taught using TGfU.

### 2.3. Procedure

#### 2.3.1. Design of Intervention Lessons

According to students’ background and setting, we decided to implement three broad and interrelated strategies built on TGfU pedagogical features (Table 2): (1) aligning the contents progression and lesson structure throughout the lessons based on principles of play; (2) including pedagogical strategies to design achievable challenging tasks; and (3) including psychosocial strategies to promote social relationships.

#### 2.3.2. Teacher’s Instruction in TGfU

To improve the teacher’s knowledge of TGfU, he was asked to attend a 30-h instruction carried out by the authors, 3 h per week for 10 weeks, following six procedures. First, we explained the pedagogical features of TGfU highlighted in this intervention (Table 2). Second, we shared insights about the students’ background and setting, rules and routines of the class structure with the teacher. Third, we showed the expected teacher’s and students’ behaviours during the intervention using TGfU. Fourth, together with the teacher, we designed 14 pilot lessons. Fifth, the teacher conducted the pilot lessons in another school, with a similar setting to the present one and these were filmed. Sixth, together with the teacher, using the footage, we checked his and the students’ behaviours in comparison to those expected and we analysed the causes of the mistakes detected based on the pedagogical features highlighted in this study. Finally, together with the teacher, the authors improved the lessons based on the mistakes observed and, subsequently, we submitted the lessons to three experts for validation. The teacher was trained for three additional hours, drawing on the importance of social relationships to enable the students to learn. The teacher had time to reflect on his physical education lessons, questioning and planning during each lesson. The authors mentored the teacher during his intervention, providing feedback on TGfU pedagogical features and clarifying his doubts at the end of each day.

#### 2.3.3. Validation the TGfU Lessons

Three blinded TGfU experts were asked to determine, quantitatively (on a scale from 1 to 5) and qualitatively, whether the lessons were designed in accordance with the three TGfU pedagogical features (Table 2). The TGfU experts were authors of renowned prestige with a publication record on TGfU in an international context and with practical experience in its implementation. The three experts rated all the features with 4 or higher and agreed that the lessons followed the pedagogical features. Nevertheless, one of the experts requested making the goals operative. Consequently, we improved the goals until we eventually attained the lessons of this study (Table 1).

#### 2.3.4. Lesson Implementation

The teacher conducted the intervention following the same pedagogical features, aims, contents and remaining lesson characteristics described (Table 1). The initial lesson plan did not change during the intervention. The intervention lasted nine weeks.

#### 2.3.5. Verifying Treatment

We used two procedures to verify that the teacher applied the lessons following the three TGfU pedagogical features. First, a TGfU expert researcher, blinded to the study aim, observed the footages of the lessons on the pedagogical features. Second, we repeated the same procedure as with the expert but using in vivo observation. Both the TGfU expert and we confirmed that the teacher achieved all the features in all the lessons.

### 2.4. Data Collection

#### 2.4.1. Game Observation

Each student was recorded for 10 min in the Lessons 1, 10 and 17, playing a game of 4 versus 4 on a 28 m × 15 m rink area. We used the game performance assessment instrument (GPAI, [27]) to assess each student’s appropriate and inappropriate decision-making, skill execution, cover and support, following the criteria described in Morales-Belando et al. [6]. The authors trained two assistant researchers, blinded to the study aim but with experience using GPAI, for 50 h, until they learned to observe the footages of the assessment games. Both observers assessed all the students in each assessment. Inter-rater reliability was obtained comparing such observations of both observers in each assessment. Observation was systematic because they assessed all the students’ game actions. The agreement between observers was calculated by percentage of agreement and intraclass correlation coefficient (ICC). The agreement between observers was between 89% and 90% (ICC > 0.95).

#### 2.4.2. Questionnaires

The students completed the three following questionnaires individually and anonymously in Lessons 1, 10 and 17. The second author explained that they were not tests and that the students should complete them in the teacher’s absence.

Knowledge: We designed an ad-hoc questionnaire from the contents addressed in the lessons and in collaboration with the teacher, following the proposal of French and Thomas [28]. The questionnaire consisted of 12 questions related to decision-making (*n* = 3), cover (*n* = 3), support (*n* = 3) and skill execution (*n* = 3), with four response options (e.g., “After passing, I have to: (a) stand still, (b) run toward the opponents’ goal, (c) run toward my goal, or (d) wait to receive a pass”). The students required about 25 min to complete the questionnaire in writing.

Enjoyment and perceived competence: Students completed the enjoyment and perceived competence scale [29]. This instrument had seven items referring to enjoying this game (e.g., “I enjoyed practicing floorball very much”, Cronbach’s α = 0.95) and feeling or considering oneself to be good at practising floorball (e.g., “I am pretty skilled practicing floorball”, Cronbach’s α = 0.98). Agreement with the items was rated on a 5-point Likert-type scale, ranging from 1 (strongly disagree) to 5 (strongly agree). The participants responded for 10 min.

Intention to be physically active: Students completed the intention to be physically active scale [30]. This instrument had five items (e.g., “after I finish the present unit, I would like to take part in floorball club training”, Cronbach’s α = 0.96). Agreement with the items was rated on a 5-point Likert-type scale, ranging from 1 (strongly disagree) to 5 (strongly agree). The participants responded for 5 min.

#### 2.4.3. Semi-Structured Interviews

The students and the teacher were interviewed twice during this study, at the end of the 8- and 14-lesson periods. Although all the interviews addressed the same topics, the focus of the questions and the language differed depending on the interviewee (students or teacher). Specifically, we organised the two focus groups of the two assessments with the students to explore their perceptions (20 participants each). The first and last authors claimed that all the students participated by means of promoting their involvement. The teacher was interviewed individually after the students’ interviews. The students’ interviews lasted approximately 60 min, and 20 min in the case of the teacher. Data were collected using a digital audio-recorder. The interview questions targeted the students and the teacher’s perceptions of the preceding lesson to identify students’ possible improvements and examine their views after each intervention period in depth. The focus groups began with pre-established questions about game performance, knowledge and psychosocial variables (e.g., students: “What did you know to do during the game?”; teacher: “Did you notice any improvement in the students’ game performance? In what sense?”). Subsequently, the moderator began a group discussion, using questions to explore the meanings of the statements in greater depth.

### 2.5. Data Analysis

#### 2.5.1. Quantitative Data

Statistical analysis of the quantitative data (decision-making, skill execution, cover, support, game performance, knowledge, enjoyment, perceived competence and intention to be physically active) was conducted using SPSS version 11.0 (IBM Corps., Armonk, NY, USA). Normality of the data was determined through the Kolmogorov–Smirnov test, which indicated that the data were nonparametric. Friedman’s χ^2^ was used to explore significant differences in the variables. Subsequently, Wilcoxon’s *Z* post-hoc comparisons were performed to determine: (a) possible significant first–second and first–third assessment differences (intra-period differences); and (b) possible significant second–third assessment differences (inter-period differences). The alpha level was set at *p* < 0.05. In addition, we calculated the effect sizes using the partial Cohen’s *w*. Given the number of students and a medium *w* [31], the statistical power of the study was 38% (α = 0.05, λ = 3.6, critical χ^2^ = 5.99).

#### 2.5.2. Qualitative Data

Students and teacher data were analysed separately, following the comparative method and a thematic analytical approach. All recorded interviews were transcribed verbatim, coded and analysed inductively. The first and last authors independently transcribed the interviews, which were cross-checked against the original recordings to ensure accuracy. They paid special attention to what students said about their experiences in the TGfU lessons. They read the transcriptions to get a sense of their scope and to detect recurring topics of emerging themes. They wrote memos during the coding process, which highlighted recurring themes, inductively clustered within sub-themes. Data were open and axial coded line-by-line and incident-to-incident descriptively, in sub-themes, which were applied to text segments. The second author again performed all the previous procedures and they discussed the themes until a consensus was achieved. Finally, the four authors engaged in a reflective dialogue, seeking accuracy and reliability.

## 3. Results

### 3.1. Quantitative Results

The quantitative results ratified the qualitative students and teacher perceptions because the students showed no statistically significant improvements between the two periods (inter-period differences, Table 3). However, there were statistically significant differences with respect to the first assessment in decision-making (χ^2^ = 30.45; *p* = 0.000), game performance (χ^2^ = 8.45; *p* = 0.015), knowledge (χ^2^ = 16.28; *p* = 0.000) and perceived competence (χ^2^ = 7.02; *p* = 0.030). Participants improved in both periods compared with the first assessment in decision-making (8-lesson period: *p* = 0.000; 14-lesson period: *p* = 0.000), game performance (8-lesson period: *p* = 0.001; 14-lesson period: *p* = 0.011) and knowledge (8-lesson period: *p* = 0.043; 14-lesson period: *p* = 0.000). Besides, they also improved after the 14-lesson period compared with the first assessment in perceived competence (*p* = 0.008, Table 3).

### 3.2. Qualitative Results

The qualitative findings showed the meaningful learning acquired by the students after the 8- and 14-lesson periods. In general, students’ comments suggested that they perceived floorball as a team game and that they realised that players had different roles and needed others to play. Furthermore, they were aware that they had to think by themselves:

*“With the defenders, we can learn from our own actions. When an opponent is dribbling, we have to push him outside the rink, moving like a crab, to prevent him from scoring”*

(student, eight-lesson period)

*“We decided what to do in the game. Before, I didn’t know where I should be located to cover and now, I know that I have to be located diagonally, defending the centre. That idea of the crab”*

(student, 14-lesson period)

*“I think that the tasks made them to think. I’m surprised that I noticed their improvement. They have improved decision-making to a great extent. By themselves, they don’t follow the ball to defend”*

(teacher, eight-lesson period)

*“They have built their own knowledge, for example, they helped each other to know how to cover”*

(teacher, 14-lesson period).

We contend that the effect of the alignment of contents progression and lesson structure, implementation of achievable challenging tasks and promotion of social relationships were the key aspects that helped to explain the similarities between the two practice volumes. Furthermore, there were interrelated elements that appeared in those key aspects: structure and relation of the lessons, contents, nature of the game, rules and the relationships among peers and with the teacher. Some of the shared students’ beliefs that illustrated this statement were:

*“We learned step-by-step because everything was interrelated. Without defenders, the game would not make sense, it would be boring and nonsensical. The rules helped us to score more”*

(students, eight-lesson period)

*“To know how to play, you have to know how to attack and defend, everything. We learned because each day, the teacher explained things and because of how he teaches”*

(students, 14-lesson period).

The teacher’s comments were in the same line:

*“Working along the same lines all the time has allowed improving decisions, as everything in the game is based on the fact that what they have been learning little by little makes sense. The questions and rules have helped a lot”*

(teacher, eight-lesson period).

*“In the shooting lesson, as one could only shoot from a game area, a student dribbled and told a teammate to put her stick in front and when she told her, the teammate lifted her stick to score. She thought up that strategy based on what we had seen in the previous lessons”*

(teacher, 14-lesson period).

Both the students and the teacher highlighted greater confidence after the 14-lesson period. That is, after the eight-lesson period, they felt a great impact and learned, whereas, after the 14-lesson period, they reinforced that learning because they had more practice time:

*“Because in the [14-lesson period], we only improved it, but where we really learned it was in the [8-lesson period]”*

(student, 14-lesson period);

*“The improvement was greater in the [14-lesson period], although in the [8-lesson period], the impact was greater because they went from nothing to everything, but they learned more with the [14-lesson period]”*

(teacher, 14-lesson period).

#### 3.2.1. Alignment of Contents Progression and Lesson Structure

The students believed that the learning progression increased their ability to understand game situations. They granted considerable importance to the content of the lessons were related so that what they practised in one lesson served them for the following one:

*“The classes of [the teacher] are related to each other, because first, he teaches us how to score and then what to do to be able to score”*

(student, eight-lesson period)

*“It’s all related. It was all together, if one day you learned something, the next day you practised it”*

(student, 14-lesson period)

*“You can see the effort we made when planning the relationship between contents, they have learned logically”*

(teacher, eight-lesson period)

*“I would prefer to use [TGfU] because it is more logical and goes lesson-by-lesson, relating contents to learn to play”*

(teacher, 14-lesson period).

Consequently, the students showed that the alignment of the structure of the lesson allowed them to learn because, initially, the teacher explained the tasks, they practised “game form”; then, they answered questions, practised “drills-for-skill development” and “returned to game form”; and, finally, they learned how to be skilful in the game. Indeed, they learned “why” before “how”, so that they transferred skills within lessons:

*“[The teacher] does not tell us when we have to lose the mark, we do it and then he asks us”*

(student, eight-lesson period)

*“[The teacher] explained the tasks to us, but he didn’t say what to do during the game, and we learned through the questions. Besides, it’s all related”*

(student, 14-lesson period).

Along the same lines, the teacher mentioned:

*“With the questions at the end you reinforce and anchor what you are working on”*

(teacher, eight-lesson period).

*“The most decisive thing is to bring the contents closer to the real situation of the game and the questions, so they understood what they have done”*

(teacher, 14-lesson period).

Furthermore, the students acknowledged that the rules helped them understand what they had to do in the game:

*“I have to intercept because that gives us 2 points and that’s why I know it’s important”*

(student, eight-lesson period).

*“In the lesson of defence of the pass, if we intercepted a pass, we summed 2 points, and that’s why we did it”*

(student, 14-lesson period).

#### 3.2.2. Implementation of Achievable Challenging Tasks

The participants shared the importance of performing tasks at an appropriate level of difficulty, focusing on a single goal per lesson. The structure of the lesson allowed them to learn little by little:

*“[The teacher] goes little by little, because we play, he asks us, we play again and he asks us until we understand it”*

(student, eight-lesson period).

*“He was going slowly so we could understand it better. When we were in the circle, he would ask us questions and then we would play to improve”*

(student, 14-lesson period).

*“Because all the tasks of each lesson were designed to learn the same thing, they gradually learned logically”*

(teacher, eight-lesson period)

*“All the time spent on planning the lessons was worth it so that they would then gradually learn logically”*

(teacher, 14-lesson period).

In particular, the students acknowledged that the rules were helpful, making it easier for them to meet the challenge in each task, which was related to the importance of the content. They had to pay attention and engage during the task presentations:

*“As each player has to cover one, it is easier and, at first, we understand why it is important”*

(student, eight-lesson period).

*“It was very important to shoot from the centre, that’s why the defenders couldn’t get into our area so it would be a little easier”*

(student, 14-lesson period).

In addition, the students commented that, in “teaching for understanding” and “review and closure”, the teacher posed questions that were easy to understand and asked them in different ways until everyone understood the reflection. The use of meaningful examples aided students to remember the key learning behaviours:

*“When we are in the circle and we do not understand, he helps us with examples. For example, the crab”*

(student, eight-lesson period).

*“For example, every day, he asks one thing and it becomes clear. If someone didn’t understand, he asked it differently”*

(student, 14-lesson period).

The teacher indicated that this was one of the most complex aspects to carry out because it was essential for all the students to understand what he intended to teach before moving on to the next lesson:

*“As we knew that they had no previous experience, the questions had to be very simple. This is one of the most difficult parts”*

(teacher, eight-lesson period).

*“The part of the questions is complex, you have to adapt them on the go and change them, first you have to know the children and be clear about what you want to teach”*

(teacher, 14-lesson period).

Finally, the contents provided them with an achievable challenge, that is, neither very easy nor very difficult, but demanding effort. They valued the importance of thinking rather than other proficiencies:

*“Before, we always repeated things and we got bored and now with [the teacher], we learn a different thing every day, we have to think and later this helps us to play”*

(student, eight-lesson period).

*“As we practised a lot, it was easy in the end”*

(student, 14-lesson period).

The teacher’s comments were consistent with the students’ comments, although he acknowledged that the assessment games were more complex than the lesson games:

*“As the level of difficulty was adapted to the students’ level, with 8 lessons, they learned many things, but in the evaluation, I could not observe those improvements”*

(teacher, eight-lesson period).

*“Although they have had to think about the game, the tasks were at their level because everyone understood them. However, the evaluation was more difficult because there were not so many rules”*

(teacher, 14-lesson period).

However, five students and the teacher acknowledged that, during the 14-lesson period, the students were bored because they had been playing the same game for nine weeks:

*“I get a little bored always playing the same game”*

(student, 14-lesson period).

*“Overall they have enjoyed themselves because they have been improving and playing more, but many of them have gotten very tired and bored”*

(teacher, 14-lesson period).

#### 3.2.3. Promotion of Social Relationships

Both the students and the teacher stated that the lessons allowed them to establish relationships with the teammates, showing that learning is not only an individual action but is also influenced by collective experiences. They performed more complex interactions because they involved meanings, expectations and emotions (relationships as members of teams):

*“We now know how to play as a team, lose and win, and not get angry if we lose. We all play together and we play as a team”*

(student, eight-lesson period).

*“If you know to dribble, but your team doesn’t know to take the ball, it’s clear that you’re going to lose, so you have to help them to learn it”*

(student, 14-lesson period).

*“I think the tasks made them think. They planned among themselves what to do”*

(teacher, eight-lesson period).

*“They have improved mostly in decision-making because they talked a lot to each other, telling the teammates where to place themselves. More in attack than in defence”*

(teacher, 14-lesson period).

These comments suggested that the students assumed that playing floorball includes the presence of others.

They also agreed in highlighting the positive relationships established with the new teacher, his personality, predisposition, concern and attention as well as his pedagogical practices:

*“He teaches us to pay more attention to him and to the rest of the teachers to be a good class and not to complain about us”*

(student, eight-lesson period).

*“We would like to continue practising because [the teacher] has made us like the game more. He was always willing to help us”*

(student, 14-lesson period).

The teacher was surprised at the positive effect of his relationship with the students:

*“Outside of the school, a father told me that his son was having a great time. Every time his son came home from physical education, he would tell him what he was doing and that it was because of me”*

(teacher, eight-lesson period).

*“I think my relationship with them was important to make them more autonomous and to like physical education”*

(teacher, 14-lesson period).

The structure of lesson and the game, in particular, were other key elements, apart from social relationships, to help their engagement and favour their perceived competence, enjoyment and intention to be physically active.

*“There are games, and we learn within the game, in the same game, every day we play and learn more things”*

(student, eight-lesson period).

*“We would like to continue receiving the classes the same way as [the teacher] uses because there are many games. We will miss this way of teaching”*

(student, 14-lesson period).

The teacher also highlighted the game’s potential to foster such attitudes:

*“I was told they had fun because they were always playing”*

(teacher, eight-lesson period).

*“They want to keep practising; what has influenced the most is the [TGfU], the game is indifferent, the model in which everyone can play is what makes them want to continue practising”*

(teacher, 14-lesson period).

Finally, unlike the teacher, only the students expressed the novelty of practising a new game (floorball) unknown to them:

*“We want to continue practising because it is a game that we have never played”*

(student, eight-lesson period).

*“I’d like to keep practising because it was a new game”*

(student, 14-lesson period).

## 4. Discussion

The purpose of this work was to explore whether fourth-grade physical education students improved their game performance, knowledge and psychosocial variables with TGfU to a greater extent after an eight-lesson period in comparison to 14-lesson period, while playing floorball. The results confirm the hypothesis, as there were no differences between the two periods. Specially, our findings, in both 8- and 14-lesson periods, were consistent with previous studies that also found improvements in short and long interventions (e.g., [4,5,6,17,19]). However, although our results are not consistent with the reviewed studies, which recommended long intervention volumes [3,11], the 14-lesson period allowed students greater time frames and therefore more reinforcement, as participants highlighted in the interviews. As a consequence, students improved in perceived competence after the 14-lesson period (Table 3). The findings of this study could support the claim that there are a multitude of conflux elements that influence the teaching–learning process, and not only the intervention volume [2,24]. In this study, the participants’ perceptions lead us to contend that the alignment of contents progression and lesson structure, the implementation of achievable challenging tasks and the promotion of social relationships were key variables to foster the meaningful learning shown by the participants in the second and third assessments.

As in previous aligned TGfU interventions, this study also showed significant improvements in both the 8- and 14-lesson periods [6,32]. In contrast, 44.45% of the studies’ variables that performed a short intervention and 17.65% that performed a long intervention, without alignment, found no improvements in decision-making, game performance and knowledge [12,15,17,20]. Alignment in TGfU is especially relevant because it allows the student to learn to play gradually, as the participants commented. We agree with the idea reported by Harvey et al. [32] that aligned practice leads to faster decision-making within the game environment because, as Bracco et al. [4] also reported, learning progressively increases the students’ ability to understand game situations. This involves knowing the students’ previous environment (curricular and extracurricular background) to establish the tactical contents to be taught and the related technical ones. Therefore, in the lessons, we designed “drills for skill development” linked to “game form” and to “return to game form”. Drawing on this alignment, through the questions in “teaching for understanding” and “review and closure”, the teacher guided the students based on their experiences in previous tasks to make them aware of their knowledge and foster their understanding. Possibly, the meaning-making could facilitate the learning of tactical knowledge. Furthermore, the questions, as we planned in the design of the lessons, also helped students to relate the contents between lessons, as the participants perceived. Seeing the relationships among pieces of information may be more important to foster understanding than merely acquiring information [9]. Finally, the rules ensured that the students would find out which were the key behaviours to learn. For this reason, we made functional and structural modifications in the “game form” and only structural modifications in the “return to game form”, so students played within an easy decision-making setting at the beginning, whereas they played within a less constrained (more difficult) setting at the end of the lessons [6].

In relation to rule constraints, the teacher acknowledged that the games in the assessment interventions were more complex than those of the lessons, as in the former there were no constraints. That lack of restrictions could reduce the students’ ability to make decisions [32]. This was also reflected in the quantitative results obtained. Whereas the participants’ statements showed the high level of meaningful learning, this was not reflected in the quantitative assessment. The differences between qualitative and quantitative results could be related to the differentiation between performance and learning [33]. In this sense, the teacher underlined that the meaningful learning was due to the care with which the tasks and questions were created, considering the students’ background. He considered this phase of the design the most difficult, as has been shown previously [34]. In the present study, basic tactical contents were selected, linking them to meaningful challenges for the students. A reflection of the relevance of the work done to select the contents was that even the students highlighted the game actions without the possession of the ball, which are more complex to learn [32]. Furthermore, aiding the learners to see the value of what they must learn could reinforce their learning in relation to their previous knowledge [11]. For, example, to defend the progression, it was explained that they could not allow the player with the ball to dribble in the centre, because they would have more passing and shooting options. To prevent the attacker with the ball from going to the centre of the rink, they had to move sideways similar to a crab. The aligned rules implemented to double score and the game in numerical superiority or inferiority could promote the expected behaviours. For this reason, the students mentioned that the tasks were an achievable challenge despite having repeated the same contents in both periods. In this sense, during the teacher’ formation, much emphasis was placed so that the reflections about “teaching for understanding” and “review and closure” could be understood by the students, and the participants acknowledged this. This understanding seems to be crucial to attain the next phase of knowledge [10]. However, after the 14-lesson period, the students stated that they were tired of playing so much floorball, as 15% of the students of Mandigo, Holt and Anderson [35] also reported.

With regard to the psychosocial variables, this work also showed significant improvements in both the 8- and 14-lesson periods in enjoyment, perceived competence and intention to be physically active as in prior studies [6,10,12,20,22]. Nevertheless, unlike previous studies, in this study, this type of variables was analysed following a mixed-method approach, which helped to explain the effect of the interaction of the interrelated elements. As the participants showed, the structure of the lessons, the use of game forms, the novelty of the content and the social relations that were established between peers and with the teacher acted as process variables to promote meaningful learning (see qualitative results). Bracco et al. [4] also found that the lesson structure, and especially the game forms, was enjoyable and led to students’ engagement. This kind of experiences could afford students the opportunities to gain confidence and the competence to be physically active in the future [36]. Specially, the students highlighted their experiences with peers [10], but also with teachers. Positive student–teacher relationships are crucial for students’ educational development (e.g., [37]). The students’ acknowledgment of the teacher’s predisposition, concern and attention towards the teaching of the contents and the development of positive behaviours was very significant and this favoured their trust in him. For example, the students indicated that they asked the teacher as many times as needed or that he helped them behave better even in other subjects, attitudes that were promoted during the teacher’s formation. Those feelings were very relevant considering the low influence of current physical education in young people’s lives, suggesting the need for different approaches that are meaningful and relevant to students [37]. The teacher’s role is especially relevant in TGfU to help students grow as learners [34]. Likewise, it seems that the students perceived peer-to-peer grouping as positive. As Koekoek and Knoppers [10] maintained, the construction of tactical knowledge benefited from the interaction between peers within the game. In this study, they assisted each other, made decisions as a group, learned values, enjoyed with their peers and collaborated within a team (see Section 3.2). The support of peers, as well as peers as necessary collaborators within a team, were also reported in Bracco et al. [4] and Koekoek and Knoppers [10].

To sum up, the interrelated nature of the overlapping elements among the variables that influenced the teaching–learning process (in this study, we highlighted the alignment of contents progression and lesson structure, the implementation of achievable challenging tasks and the promotion of social relationships) provides further evidence that the social environment is a key aspect in such process. This context permeated what and how students learn [8]. The greater the teacher’s ability to take such variables into account, the greater the consistency of the intervention in relation to the environment and the more powerful the students’ learning. That could be why some TGfU studies in which a long unit was implemented, based on such elements, showed negative results [16,19,20], whereas other studies with a short unit and considering these elements reported positive results [6,17,23].

## 5. Conclusions

According to the quantitative and qualitative results, the amount of practice should not be considered as the only variable in the design of interventions with TGfU. The qualitative results supported that the pedagogical strategies implemented could be key to explain the similarities between the two practice volumes. Considering that the educational curriculum requires short-term interventions, we recommend paying special attention to the interaction between the alignment of contents progression and lesson structure, the implementation of achievable challenging tasks and the promotion of social relationships according to students’ background. In other words, TGfU interventions should not separate the length of the unit from the TGfU pedagogical features (Table 2). Those variables could help students to make decisions with sense and therefore, foster meaningful learning, as this study showed. Nevertheless, the results should be interpreted with caution because there was no control group, the small number of participants, the lower statistical power, the sensitivity of GPAI, the possible familiarisation with the testing procedures and the possible social influence in the students’ perceptions during the focus groups. Studies such as the present, focused on how TGfU pedagogical features should be assembled, are necessary to show a guideline/template to help the teachers to implement TGfU.

## Figures and Tables

**Table 1:** Features of the Teaching Games for Understanding Unit.

Lesson and Tactical Principle (Goal)	Tactical-Technical Content	Game Form	Teaching for Understanding	Drills for Skill Development	Return to Game Form	Review and Closure
First assessment	-	4 vs. 4; 28 × 15 m.	-	-	-	-
Shooting on goal	- Giving priority to shooting over passing and running with the ball. Shooting from the centre of the rink. - Shooting follow through the movement from the back leg to the front foot.	3 vs. 2; 10 × 5 m; double score if an attacker on-the-ball shoots from a central rink area; forbidden to pass more than three times; compulsory man-to-man defence.	Once you get the ball, what is the first game action to do? Which area of the rink allows you to shoot on goal more easily? Why?	Shooting using the heel of the stick facing the goal, changing the goalkeeper after each shot.	3 vs. 2; 10 m × 5 m; triple score if an attacker on-the-ball shoots from the centre rink.	When should you shoot on goal? How should you move your body to score? Why?
Defending the goal	- Defending close to an attacker on-the-ball who is going to shoot (locating between the attacker on-the-ball and the goal). - Bending the knees, striding and adjusting to defend the shot on the goal.	2 vs. 2; 8 × 4 m; double score if the defence avoids the shot; forbidden to defend on the shot area (restricted area where only an attacker on-the-ball can shoot) if there is no attacker in that area.	How did you hinder an attacker’s shot? Where should you be, far or close to an attacker on-the-ball?	Trying to intercept the shots, bending the knees, striding and adjusting to defend an attacker on-the-ball shooting on goal.	2 vs. 2; 8 m × 4 m; triple score if a defender intercepts a shot.	Where should you be located to defend the goal? Why? How should you position your body?
Possession of the ball	- Receiving the pass leaving the defence behind. - Holding the stick with both hands (the dominant hand closest to the ball); not raising the stick above the knees.	4 vs. 3; 10 × 5 m; double score if an attacker on-the-ball passes to a teammate, then progresses to goal and finally gets back the ball for a shot to the goal; forbidden to intercept the pass; compulsory man-to-man defence.	What can you do to receive a pass? How can you support your teammate on-the-ball? What teammate do you have to pass the ball to?	Passing holding the stick with the dominant hand closest to the ball; not raising the stick above the knees.	4 vs. 3; 10 m × 5 m; triple score if an attacker on-the-ball passes to a teammate, then progresses to goal and finally gets back the ball for a shot to the goal.	Where should you be located to receive the pass? Why? How should you hold the stick to pass to a teammate?
Winning the ball	- Defending your attacker off-the-ball located in the centre of a triangle formed by the goal, the attacker on-the-ball and the attacker off-the-ball. - Bending the knees and extending the stick to intercept the pass.	4 vs. 4; 20 × 10 m; double score if a defender intercepts the pass of an attacker off-the-ball; forbidden to pass to the nearest player or to the one who just made the pass; compulsory man-to-man defence and defensive help from an attacker on-the-ball.	What did you do to hinder an attacker to pass and receive? Where should you be located?	Trying to intercept passes bending the knees and extending the stick.	4 vs. 4; 20 m × 10 m; triple score if a defender intercepts a pass.	Where should you be located to win ball possession when you are defending an attacker off-the-ball? Why? How should you position your body and stick to get the ball?
Attacking the goal	- Dribbling from the sides of the rink to attack the goal. Supporting an attacker on-the-ball to receive a pass. - Dribbling without looking at the ball.	4 vs. 3; 10 × 5 m; double score in a shot after dribbling on the sides; forbidden to win the ball on the rink sides; forbidden to shoot the ball from the sides of the rink; compulsory dribbling on the rink sides.	What rink location is easier for dribbling? Why? What can you do to receive a pass from the side area? Where do you have to be located to receive that ball, close or far from an attacker on-the-ball?	Dribbling looking ahead from one end of the rink to the other.	4 vs. 3; 10 m × 5 m; triple score if the attacker on-the-ball shoots to the goal after dribbling on the rink sides. Triple score in a shot after a pass and dribbling on the sides.	Where should you be located to dribble the ball? Why? How do you support the attacker on-the ball dribbling? How should you dribble (looking at the ball or without looking at it)?
Challenging the opponents’ progression	- Defending an attacker on-the-ball, leaving some space to react if he/she is going to dribble. - Body perpendicular to the goal to keep an attacker on-the-ball on the sides of the rink.	2 vs. 2; 8 × 4 m; double score if a defender recovers the ball on the rink sides; compulsory dribbling on the rink sides; compulsory to win the ball on the rink sides.	How can you challenge the opponents’ progression? Where are you positioned, close or far from an attacker on-the-ball? Why?	Trying to keep an attacker on-the-ball on the sides of the rink, positioning the body perpendicular to the goal.	2 vs. 2; 8 m × 4 m; triple score if a defender wins the ball from an attacker on-the-ball on the rink sides.	What should you do to hinder an attacker on-the-ball’s progression? Why? How should you position your body to keep an attacker on the sides of the rink?
Attacking the goal	- Changing the pace and direction to lose the mark of a defender. - Changing the pace and direction at the same time to lose the mark of a defender.	4 vs. 3; 10 × 5 m; double score if you lose the mark of your defender and shoot on goal; forbidden for another defender to intercept the pass; compulsory man-to-man defence.	What should you do when a teammate has the ball? Where should you be to receive the pass of a teammate?	Trying to lose the mark of a defender changing the pace and direction at the same time.	4 vs. 3; 10 m × 5 m; triple score if you lose the mark and shoot on goal.	What should you do to lose the mark from your defender? How should you lose the mark from your defender?
Challenging the opponents’ progression	- Defending close to your attacker off-the-ball who is trying to lose the mark. - Extending the arm and the stick to deny the passing lane.	4 vs. 4; 20 × 10 m; double score if a defender of an attacker off-the-ball intercepts or denies the pass; forbidden to dribble and to steal the ball.	How can you defend an attacker off-the-ball? What should you take into consideration to adjust your location?	Trying to deny the passing lane, extending the arm and the stick.	4 vs. 4; 20 m × 10 m; triple score if the defence of an attacker off-the-ball intercepts or denies the pass.	Where should you be located to defend your attacker off-the-ball who is trying to lose the mark? How should you position your arm and stick to deny the passing lane?
Second intervention assessment	-	4 vs. 4; 28 × 15 m.	-	-	-	-
Shooting on goal	- Shooting from the centre of the rink. - Shooting follow through the movement from the back leg to the front foot.	4 vs. 3; 10 × 5 m; double score if an attacker on-the-ball shoots from a central rink area; compulsory man-to-man defence.	Which area of the rink allows you to shoot on goal more easily? Why?	Shooting using the heel of the stick facing the goal, changing the goalkeeper after each shot.	4 vs. 3; 10 m × 5 m; triple score if an attacker on-the-ball shoots from the centre rink.	Where should you shoot on goal? How should you move your body to score? Why?
Defending the goal	- Defending located between the attacker on-the-ball and the goal. - Bending the knees, striding and adjusting to defend the shot on the goal.	3 vs. 3; 10 × 5 m; double score if the defence avoids the shot; forbidden to defend on the shot area (restricted area where only an attacker on-the-ball can shoot) if there is no attacker in that area.	How did you hinder an attacker’s shot?	Trying to intercept the shots, bending the knees, striding and adjusting to defend an attacker on-the-ball shooting on goal.	3 vs. 3; 10 m × 5 m; triple score if a defender intercepts a shot.	Where should you be located to defend the goal? Why? How should you position your body?
Maintaining possession of the ball	- Receiving the pass leaving the defence behind. - Holding the stick with both hands (the dominant hand closest to the ball).	3 vs. 2; 10 × 5 m; double score if an attacker on-the-ball passes to a teammate, then progresses to goal and finally gets back the ball for a shot to the goal; compulsory man-to-man defence.	What can you do to receive a pass? How can you support your teammate on-the-ball? What teammate do you have to pass the ball to?	Passing, holding the stick with the dominant hand closest to the ball; not raising the stick above the knees.	3 vs. 2; 10 m × 5 m; triple score if an attacker on-the-ball passes to a teammate, then progresses to goal and finally gets back the ball for a shot to the goal.	Where should you be located to receive the pass? Why? How should you hold the stick to pass to a teammate?
Winning the ball	- Defending your attacker off-the-ball located in the centre of a triangle formed by the goal, the attacker on-the-ball and the attacker off-the-ball. - Bending the knees and extending the stick to intercept the pass.	3 vs. 3; 10 × 5 m; double score if a defender intercepts the pass of an attacker off-the-ball; compulsory man-to-man defence and defensive help from an attacker on-the-ball.	What did you do to hinder an attacker to pass and receive? Where should you be located?	Trying to intercept passes bending the knees and extending the stick.	3 vs. 3; 10 m × 5 m; triple score if a defender intercepts a pass.	Where should you be located to win ball possession when you are defending an attacker off-the-ball? Why? How should you position your body and stick to get the ball?
Attacking the goal	- Supporting an attacker on-the-ball to receive a pass. - Dribbling without looking at the ball.	3 vs. 2; 10 × 5 m; double score in a shot after dribbling on the sides; forbidden to win the ball on the rink sides; compulsory dribbling on the rink sides.	What can you do to receive a pass from the side area? Where do you have to be located to receive that ball, close or far from an attacker on-the-ball?	Dribbling looking ahead from one end of the rink to the other.	3 vs. 2; 10 m × 5 m; triple score if the attacker on-the-ball shoots to the goal after dribbling on the rink sides. Triple score in a shot after a pass and dribbling on the sides.	How do you support the attacker on-the ball dribbling? How should you dribble (looking at the ball or without looking at it)?
Challenging the opponents’ progression	- Defending an attacker on-the-ball, leaving some space to react if he/she is going to dribble. - Body perpendicular to the goal to keep an attacker on-the-ball on the sides of the rink.	3 vs. 3; 10 × 5 m; double score if a defender recovers the ball on the rink sides; compulsory dribbling on the rink sides.	How can you challenge the opponents’ progression? Where are you positioned, close or far from an attacker on-the-ball? Why?	Trying to keep an attacker on-the-ball on the sides of the rink, positioning the body perpendicular to the goal.	3 vs. 3; 10 m × 5 m; triple score if a defender wins the ball from an attacker on-the-ball on the rink sides.	What should you do to hinder an attacker on-the-ball’s progression? Why? How should you position your body to keep an attacker on the sides of the rink?
Third intervention assessment	-	4 vs. 4; 28 × 15 m.	-	-	-	-

**Table 2:** Strategies to Design the Lessons Built on Teaching Games for Understanding Pedagogical Features and Examples (See More Details about Each Strategy in Table 1).

**Strategies to Promote the Alignment of Contents Progression and Lesson Structure [6,25]**	**Example**
1. We selected the teaching–learning contents of each lesson and their progression based on the principles of play, following the criteria of motivation, tactical priority and complexity.	The progression of the contents was: shooting on goal (e.g., giving priority to shooting over passing and running with the ball), possession of the ball (e.g., receiving the pass leaving the defence behind) and attacking the goal (e.g., dribbling from the sides of the rink to attack the goal). First, focusing on the attack and later in the defence.
2. The tactical and technical contents, as well as the tasks, questions, rink spaces, number of players and remaining rules were aligned with this lesson goal. Therefore, each lesson was contextualised in a principle of play, which made it possible to establish its objective.	In the sixth lesson, the principle of play was to attack the goal. Accordingly, the students should have understood that they should dribble from the rink sides to attack the goal and support an attacker on-the-ball (tactical content). Then, they had to learn how to dribble without looking at the ball (technical content). Consequently, game forms were designed with more attackers than defenders; larger rink spaces; questions related to understanding where, when, what, why and how to dribble; forbidden and compulsory rules; and double score for supporting the expected behaviour.
3. We designed the lessons in collaboration with the teacher and following the same structure.	In “game form”, students practised decision-making in a much constrained game form, using functional and structural modifications (size of the rink, value of the goals, forbidden and compulsory game actions, kind of defences). In “teaching for understanding”, they reflected on what they had to do and why with regard to the previous tasks and the previous lessons, through teacher’s questions to make them aware of their knowledge and foster their understanding. In “drills for skill development”, the students practised the technical content related to the tactical content. In “return to game form”, they performed a similar task to the initial one, but less constrained, using structural modifications (size of the rink, value of the goals). In “review and closure”, the students again reflected, this time, on the integration and understanding of decision-making and skill execution.
**Strategies to Promote the Implementation of Achievable Challenging Tasks [11,26]**	**Example**
1. We chose basic tactical and technical contents appropriate to the students’ developmental stage to promote exploratory behaviour.	In the second lesson, they had to learn to give priority to shooting over passing and running with the ball.
2. We focused each lesson on a single goal to prevent students’ dispersion.	In the third lesson, the aim was to defend the goal. Consequently, they had to learn where to be located and how they should position their body.
3. The teacher started each lesson emphasising the value and significance of the content related to previous contents to be able to play the game.	In the fourth lesson, the teacher explained that passing the ball (new content) was so important to achieve a good shooting position (previous content).
4. The teacher used meaningful examples to foster the relationship between students’ previous experience and new knowledge.	In the seventh lesson, the teacher explained that, to prevent the attacker with the ball from going to the centre of the rink, students should move like a crab.
5. Although the time of each task was pre-established, the teacher was free to use more time in “teaching for understanding” and “review and closure” to favour all the students’ understanding.	The time of effective practice was 15, 10 and 15 min in “game form”, “drills for skill development” and “return to game form” tasks, respectively. The students were only physically inactive during the tasks’ explanations (2 min) and in “teaching for understanding” and “review and closure” (8–10 min each).
6. Drawing on the pre-established questions in “teaching for understanding” and “review and closure”, the teacher could introduce new questions adapted to students’ answers.	In the fifth lesson, drawing on the pre-established questions (“What did you do to hinder an attacker to pass and receive?” and “Where should you be located?”), the teacher added: Should you be close to the attacker-on-the ball? Should you be close to your attacker-off-the ball? Should you be close to the goal?
**Strategies to Promote the Social Relationships [4,5,10]**	**Example**
1. We chose floorball as a new team game because it requires students to focus on group interactions and relationships to learn.	In the eleventh lesson, the students related each other to develop strategies in order to trick the defenders and to score (teacher commented: “In the shooting lesson, as one could only shoot from a game area, a student dribbled and told a teammate to put her stick in front and when she told her, the teammate lifted her stick to score. She thought up that strategy based on what we had seen in the previous lessons”).
2. Students participated in tasks within heterogeneous groups (by gender) of two to four players.	Along the lessons, the matches varied from 2 vs. 2 to 4 vs. 4.
3. The teacher created new group compositions for each lesson.	The teacher assigned a number for each student and he randomly assigned each student to a different group.
4. The teacher asked questions in “teaching for understanding” and “review and closure” to the whole group standing in a semicircle.	The teacher determined the centre of the rink as the pre-established area to ask the questions.
5. During teacher training, the teacher was encouraged to provide a more personal and caring attention, building students’ motivation and positive attitudes.	The teacher was very close to the students to interact with all of them during each lesson. He encouraged them to try different solutions and share ideas with their peers. The teacher used the following statements: “If you have doubts, you can ask me as many times as you need”; “I think you are able to do… talking with your teammates”; “I trust you”; “It does not matter if you make mistakes. The mistakes help us to learn”; “You are very focused on the task”; “I am so happy to see you improving”; and “You are doing very well”.

**Table 3:** Medians, Interquartile Ranges and Significant Differences of the Variables at the 8- and 14-Lesson Periods.

Variables	Intra-Period Differences							Inter-Period Differences		
	First Assessment	Second Assessment (8-Lesson Period)			Third Assessment (14-Lesson Period)					
	Mdn (IQR)	Mdn (IQR)	*p*	*w*	Mdn (IQR)	*p*	*w*	Z	*p*	*w*
Decision-making	0.00 (0.00,0.12)	0.39 (0.16,1.00)	0.000 *	1.32	0.29 (0.00,0.66)	0.000 *	0.83	1.13	0.258	0.07
Skill execution	0.50 (0.01,1.00)	0.46 (0.00,1.25)	0.527	0.35	0.90 (0.33,1.62)	0.105	0.49	0.82	0.408	0.02
Cover	0.25 (0.12,0.36)	0.25 (0.11,0.44)	0.879	0.21	0.26 (0.08,0.40)	0.873	0.08	0.60	0.544	0.13
Support	0.09 (0.00,0.22)	0.17 (0.00,0.34)	0.059	0.50	0.09 (0.00,0.29)	0.837	0.09	1.54	0.124	0.42
Game performance	0.27 (0.12,0.47)	0.40 (0.25,0.77)	0.001 *	0.84	0.42 (0.23,0.74)	0.011 *	0.83	0.01	0.989	0.07
Knowledge	5.00 (4.00,6.00)	7.00 (5.00,8.00)	0.043 *	0.56	7.00 (6.00,8.00)	0.000 *	1.22	1.77	0.075	0.47
Enjoyment	4.83 (4.50,5.00)	4.91 (4.66,5.00	0.779	0.15	4.66 (4.50,5.00)	0.183	0.36	2.08	0.058	0.52
Perceived competence	4.00 (3.75,4.68)	4.37 (4.00,4.93)	0.115	0.36	4.5 (4.00,5.00)	0.008 *	0.68	1.26	0.207	0.32
Intention to be physically active	4.80 (4.20,5.00)	4.80 (4.60,5.00)	0.171	0.37	4.80 (4.45,5.00)	0.176	0.36	0.37	0.704	0.06

* Statistical significant differences *p* < 0.05. Mdn, median; IQR, interquartile range; *w*, effect size.
